# Influence of Vigilance Performance on Lifeguard Gaze Behaviour

**DOI:** 10.5964/ejop.12121

**Published:** 2024-08-30

**Authors:** Benjamin T. Sharpe, Jenny Smith

**Affiliations:** 1 Institute of Psychology, Business, and Human Sciences, University of Chichester, Chichester, United Kingdom; 2 Institute of Applied Sciences, University of Chichester, Chichester, United Kingdom; Università degli Studi di Bari Aldo Moro, Bari, Italy

**Keywords:** lifeguard, expertise, drowning detection, vigilance, gaze behaviour

## Abstract

The present study sought to examine the gaze behaviours exhibited by lifeguards with different levels of experience while performing a task focused on detecting drowning incidents across extended periods. The results indicated a gradual decline in detection performance over time, regardless of the lifeguards' levels of experience. Analysis of the participants' gaze behaviours unveiled that this decline was associated with alterations in both the number and duration of fixations. The results indicated that lifeguards with greater experience maintained higher levels of detection performance and fixation numbers for extended durations, while exhibiting consistent fixation durations throughout the task, in contrast to their less experienced counterparts. These findings offer initial indications that lifeguards with more experience may possess an attentional advantage during tasks requiring sustained vigilance.

A lifeguard's proficiency in processing visual information is crucial for ensuring bather safety, encompassing tasks like monitoring swimmer behaviors, anticipating potential hazards, and responding promptly to drowning scenarios (Hunsucker & Davison, 2008; Lanagan-Leitzel, 2012; Petrass & Blitvich, 2014), over prolonged time frames (Schwebel et al., 2011). The extended nature of a lifeguard task, and the ability of a lifeguard to process such multitude of information over time (i.e., bathers of different swim capacity), is referred to as a vigilance task. Vigilance is defined as the ability to sustain attention and remain alert to a specific stimulus over an extended period (Davies & Parasuraman, 1982; Hirter & Van Nest, 1995; Parasuraman & Davies, 1977; Warm & Parasuraman, 1987), and is a relatively unexplored facet of lifeguarding research despite lifeguards potentially spending up to sixty minutes continuously observing an aquatic scene (RLSS, 2017). This knowledge gap raises concerns for bathers and lifeguard organizations, especially considering evidence indicating increased attentional lapses during prolonged monitoring (e.g., Killingsworth & Gilbert, 2010; Sharpe & Hale, 2023). The decline in ability associated with extended monitoring, termed vigilance decrement (Warm & Parasuraman, 1987), is identified as a leading cause of occupational incidents (Edkins & Pollock, 1997). Consequently, this study aims to address this gap by examining the gaze behaviors of lifeguards with varying experience levels during a specific vigilance task focused on detecting drownings.

Early studies (e.g., Nuechterlein et al., 1983; Parasuraman & Davies, 1977; Teichner, 1974; Warm et al., 2008) and more recent literature (Jackson & Balota, 2012; Molloy & Parasuraman, 1996; Sharpe & Hale, 2023; Unsworth et al., 2010, 2020) show that target detection performance decreases during a monotonous task. Such investigation into lifeguard vigilance, from a theoretical perspective, may provide insight into our human capacity to maintain attention and the systems responsible for the failure of such attention. Particularly when a lifeguard’s role is to maintain optimal efficiency over the duration of their shift. The role has similarities with other occupation (e.g., vehicle operation, air traffic control, military surveillance, etc.) where a vigilance decrement has been observed after only eight to thirty minutes (Molloy & Parasuraman, 1996; Verster & Roth, 2013). It may be suggested that these decrements are due to task underload (e.g., monotony) or the nature of continuously processing of stimuli resulting in task overload (e.g., cognitive resource depletion). Specifically, the underload account proposes lapses in attention are caused from tasks being under-stimulating (Helton & Russell, 2015; Manly et al., 1999), whilst the overload account suggests attentional lapses likely increase when the task at hand is objectively more challenging (Grier et al., 2003; Helton et al., 2005).

A state of cognitive underload is common during continuous, monotonous, and low demand scenarios. According to the theory of attentional resource shrinkage (Young & Stanton, 2007), a cognitively underloading task may reduce attention and, subsequently, lead to a vigilance decrement in detection performance. Ma et al. (2018) found that drivers who experienced low variability of vehicle speed reported less vigilance than drivers in a large variability group. When lifeguarding, variability of drowning duration, bather number, and task length also varies substantially. However, in scenarios where bather numbers are constant (e.g., swimming lessons), underload could lead to a reduction in vigilance and drowning detection performance. In the field of lifeguarding research, the question remains unresolved as to whether continuous, monotonous, and low-demand scenarios yield results that align consistently or show a tendency in line with prior eye tracking focused studies in the literature. It would be anticipated, however, that under such condition novice lifeguard would remain less likely to detect a drowning victim compared to their more experienced counterparts (Page et al., 2011), have slower response times to scenarios (Laxton, Crundall, et al., 2021; Laxton, Guest, et al., 2021), and distracted by irrelevant aspects of a display (Vansteenkiste et al., 2021).

Contrary to the notion that the lifeguard role is inherently monotonous, it is plausible that lifeguards perceive their responsibilities as increasingly challenging, contingent upon contextual factors such as rising bather numbers or the inherent anxiety associated with preventing drowning incidents. Drawing from Hockey's compensatory control model (Hockey, 1997), which posits that individuals adjust their effort in response to the perceived importance of goals, variations in mental effort are likely to correspond to task difficulty. As such, a high stakes and busy aquatic environment may result in increased mental effort. In scenarios characterized by increased task difficulty, particularly in stressful multitasking situations, there is a risk of excessive utilization of cognitive resources, potentially diminishing overall cognitive ability (Esterman et al., 2014; Head & Helton, 2015). Lifeguards may encounter cognitive overload when confronted with busy scenes containing numerous potential hazards (i.e., high task demand), a situation exacerbated by the engagement of endogenous top-down control. However, resource control theory posits that our ability to regulate cognitive resources over time reflects the extent of vigilance decrement experienced (Thomson et al., 2015). While the current study does not aim to test these theoretical predictions directly, gaining insights into lifeguard responses across varying display complexities over extended durations may aid researchers in interpreting findings related to cognitive resource allocation and vigilance decrement.

Eye movements have been employed to assess vigilance, as prior research suggests a potential correlation between sustained attention and eye movements (Bodala et al., 2017). Naeeri et al. (2019) observed pilots exhibiting fewer fixations of longer duration in the final hour of a four-hour task, while Bodala et al. (2017) noted a substantial decrease in saccade amplitude and velocity with vigilance decrement. Conversely, Saito (1992) found no significant quantitative changes in saccadic eye movement during a five-hour eye-tracking task. Some studies indicate that blink frequency (McIntire et al., 2014) and blink duration increase with vigilance decrement (Häkkänen et al., 1999). In the context of lifeguarding, Vansteenkiste et al. (2021) employed a vigilance-based 45-minute task but did not examine changes in eye movements over the duration. Notably, to the best of the authors' knowledge, no other studies have analysed time-based differences in eye movements in lifeguarding beyond a 20-minute timeframe. Irrespective, prior research on lifeguard experience has revealed consistent differences in gaze behaviour among lifeguards of different experience levels in short duration tasks (Lanagan-Leitzel & Moore, 2010; Vansteenkiste et al., 2021). For example, experienced lifeguards demonstrate more fixations than novices (Lanagan-Leitzel & Moore, 2010), and beach lifeguards with experience maintain longer fixations on task-relevant stimuli than their novice counterparts (Vansteenkiste et al., 2021). However, some studies suggest that eye movements may not consistently distinguish lifeguard performance differences associated with experience (Laxton, Crundall, et al., 2021; Laxton et al., 2022; Laxton, Guest, et al., 2021; Page et al., 2011; Smith et al., 2020). Task variations in representativeness, complexity, and duration have been proposed as potential contributors to these contradictory findings (Sharpe et al., 2023; Smith, 2016). As such, the following study will adopt a simulated task that has been demonstrated to elicit expertise effects in a controllable scenario, with the hopes that future researchers will extend our work into in-situ environments.

## Aims

The present study aims to investigate the gaze behaviours of lifeguards with varying levels of experience during a specific vigilance task focused on detecting drownings. Based on previous literature (Laxton, Crundall, et al., 2021; Laxton, Guest, et al., 2021; Page et al., 2011), we hypothesize that experienced lifeguards would outperform those with less experience. Additionally, we predict that participants would experience a decline in vigilance during the task, as supported by previous studies (Sharpe et al., 2023, 2024; Verster & Roth, 2013). We further anticipate that differences in detection performance would be related to changes in gaze behaviour. Specifically, we expect that experienced lifeguards would exhibit fewer fixations of longer duration and shorter blink durations compared to their less experienced counterparts (McIntire et al., 2014; Vansteenkiste et al., 2021).

## Method

### Participants

A total of 108 participants aged 18 to 36 years (*M age =* 24.5, *SD =* 5.7 years), consisting of 36 females and 72 males, took part in the study. Originally, there were 111 participants but three were removed due to corrupt eye tracking data. The criteria for grouping were established using a methodology consistent with previous authors (Page et al., 2011; Sharpe et al., 2023). Those with greater than 100 months of lifeguarding experience were considered *experienced* and those with 12 months or less were considered *novice*, lifeguards between this criterion were considered *intermediate*. 20 were considered experienced (*M* lifeguard employment = 182.45, *SD* = 40.46 months), 47 were considered intermediate (*M* lifeguard employment = 42.38, *SD* = 29.02 months), and 41 were considered novice (*M* lifeguard employment = 5.22, *SD* = 3.49 months). At the time of the study, all lifeguards were actively employed across a range of lifeguarding roles. The sample included beach (private = 20, public = 22, surf = 12) and poolside (recreational = 44, private = 6, competitive = 4) lifeguards. An *a priori* power analysis was conducted using G*power (Faul et al., 2007). Due to our interest in the interaction between expertise and vigilance performance, we targeted the within-between interaction in a repeated-measures ANOVA and based our calculations on the experience by vigilance interaction effect size ($\eta_{p}^{2}$ = 0.235) reported by Sharpe et al. (2023) who measured performance by experienced and less-experienced lifeguards in a drowning detection vigilance task. As such, for a power (1-β) of .95 and a two-tailed α of .05, the minimum sample size across the three groups was *n* = 57. Ethical approval for the study protocol was awarded by the lead institution. All participants provided informed consent prior to the onset of the data collection.

### Instruments

#### Bobbing Along Performance Task

**Figure 1:**
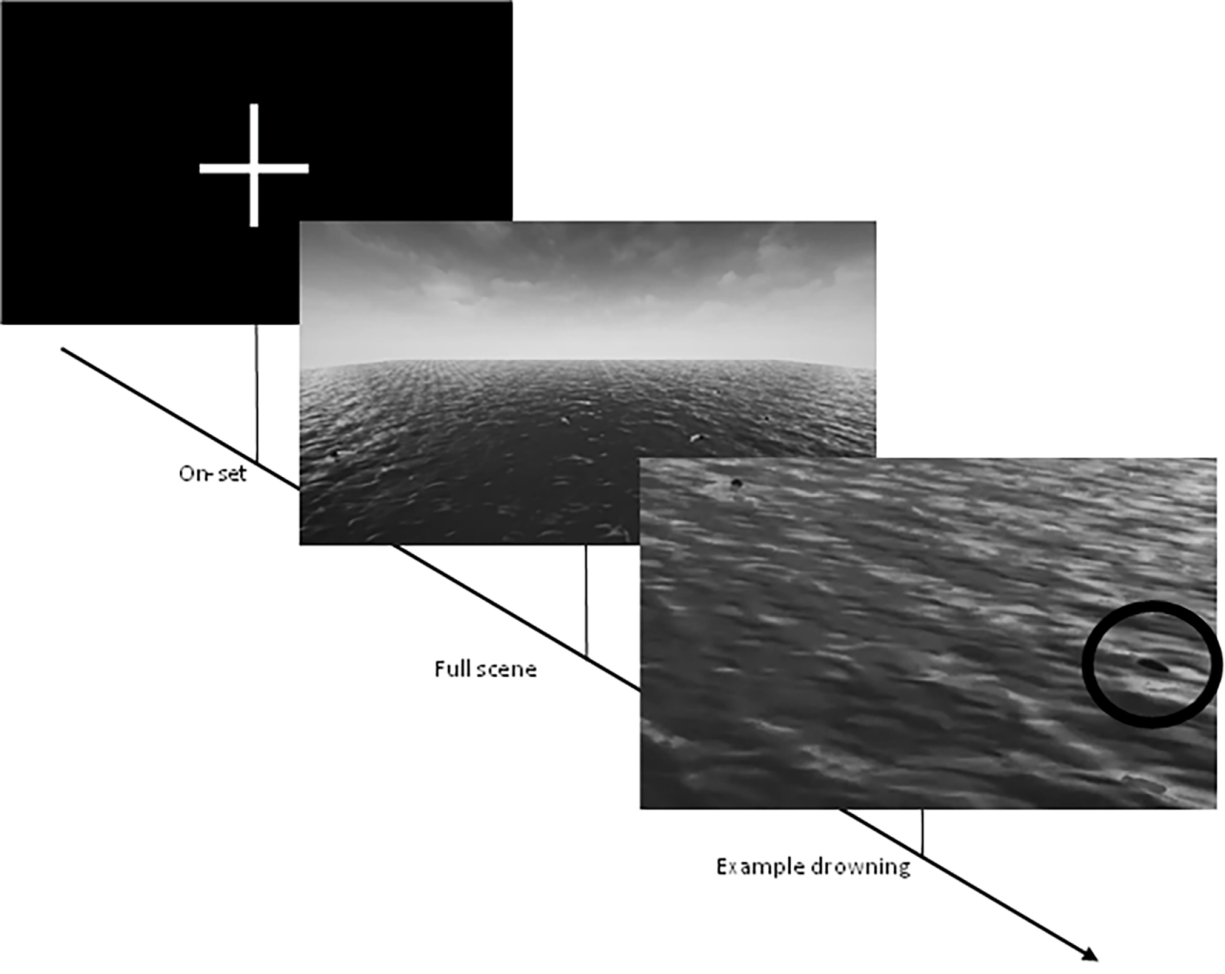
Bobbing Along Performance Task *Note.* On-set demonstrates the moment prior to the initiation of the task. Black circle indicates an example of a drowning event.

The study utilized a drowning detection tool called "Bobbing Along" initially presented by Sharpe et al. (Sharpe et al., 2023; Sharpe et al., 2024; see Supplementary Materials for task preview). This toosl specifically targets a lifeguard’s responsibility to detect drowning scenarios and simulates the maximum recommended duration that a certified lifeguard may encounter during their duty, adhering to the guidelines set by the Royal Life Saving Society (RLSS UK) which suggests a task duration of up to sixty minutes (RLSS, 2017). The environment was divided into 16 navigation meshes, with two AI-controlled actors ("bathers") assigned to each mesh. These actors moved in a randomized manner within their respective meshes, simulating swimming behaviour. In the event of a "drown" occurrence (see Figure 1), a designated bather would gradually submerge over a span of 30 seconds while treading water. Importantly, the sixty-minute task did not restart, pause, or reset the positions of the bathers. Once a bather fully submerged, they resurfaced and resumed their randomized swimming pattern. This continuous nature of the task aimed to replicate the real-world context of a lifeguard's responsibility, which involves monitoring all individuals within an aquatic space.

#### Gaze Behaviour

The relative position of the pupil and corneal reflection (i.e., gaze behaviour) was measured by a head-mounted 50 Hz four-sensor Tobii Pro 2.0 mobile eye tracker. Operated via Tobii Pro Glasses Controller Software, the system recorded the point-of-gaze based on binocular corneal reflection with respect to an integrated camera. The integrated view camera recorded 1920 x 1080 at 25fps with a 90˚ field of view. Horizontal and vertical system accuracy was considered appropriately calibrated when system accuracy reached 0.5º precision. The head-mounted eye tracker held an 82˚ horizontal and 52˚ vertical field of view. Eye data (i.e., fixations and blink rate) was analysed using the Tobii Pro Lab Analyzer. A fixation was defined as a gaze that was maintained on a location within 1° of visual angle for a minimum of 120ms (Vickers, 1996; Vickers & Williams, 2007). Blink duration (i.e., occlusion of the pupil by the eyelid) was recorded automatically when the pupil was 80% occluded or greater for more than 120ms (see Cori et al., 2019 for review).

### Procedure

All testing procedures were conducted during regular working hours, specifically from 7 am to 3 pm. Prior to the commencement of the tasks, participants were requested to complete a consent form and a demographic questionnaire, which collected information such as age, gender, and lifeguarding experience. To ensure participants' understanding of the target stimuli, specifically the occurrence of a drowning event, they were provided with a practice trial. This trial aimed to ensure participants could clearly perceive the display and comprehend the nature of the stimuli. Adequate time was allocated for participants to ask any questions they had and find a comfortable seating position. Participants were instructed to indicate their detection of a drowning event by responding promptly. This response was recorded using a clicker device, allowing the researcher to accurately capture the timing of each event detection. Participants were encouraged to make multiple responses and verbally express their decisions. A researcher was present throughout the testing sessions to ensure the validity of the detections, ensuring that participants did not mistakenly respond to false alarms during actual drowning events (i.e., false positives). Due to researcher constraints (i.e., cognitively demanding data recording process during the on-going task), false alarm data was not collected and stored. Each task consisted of eleven drowning events and based on the number of successful detections (Hits = 1, Misses = 0), the researchers calculated a performance score ranging from zero to eleven.

The task was presented 2m away from the participant on a 16ft x 9ft high definition (4K) SAMSUNG widescreen 16:9 projector via an ASUS gaming computer (GEFORCE GTX 980). Unbeknownst to the participants, a series of drowning events occurred at exact five-minute intervals within a predetermined location, encompassing a total of 11 incidents (see supplemental figures). The drowning locations were selected randomly, adopting a non-linear sequence (e.g., back middle, front left, middle right, etc.), and did not adhere to a straightforward progression (e.g., front, middle, and then back). Each participant observed an identical iteration of the task, and they remained unaware of the total number of drowning events taking place during the experiment. Although the durations of the drowning events potentially followed a discernible pattern, none of the participants reported recognizing such a pattern, nor did the collected data indicate any noticeable trends in this regard. Each participant participated in the task alone, in a quiet, and artificially lit room. The room remained darkened from natural light so that illumination could be controlled (*M* _Horizontal_ = 11.34, *SD* = 3.69 Lx; *M*_Vertical_ = 42.09, *SD* = 6.11 Lx) across all testing (recorded through the LUX LIGHT APP).

### Statistical Analysis

Prior to conducting the analyses, the data for each observed variable were subjected to screening procedures to assess univariate normality. Skewness and kurtosis ratios, as recommended by Fallowfield et al. (2005), were used as indicators. Results indicated that skewness and kurtosis values for all measures satisfied the normality criteria outlined by Kline (1998). Outliers in the data were examined using boxplots, and no univariate or multivariate outliers were identified in the dataset. Following these preliminary steps, two-way mixed design ANOVAs were employed to examine the effects of lifeguard experience (Expert, Intermediate, Novice) and time (11 drowning scenarios) on various variables, including drowning detection performance (i.e., the number of correct Hits), number of fixations, fixation duration (i.e., fixations lasting over 100 ms), and blink duration (i.e., occlusion of the pupil). To account for multiple comparisons and minimize the risk of Type I errors (McLaughlin & Sainani, 2014), a Bonferroni adjustment was applied, which reduced the significance threshold. Additionally, violations of sphericity were addressed by adjusting the degrees of freedom using the Greenhouse-Geisser correction when epsilon was below 0.75 and the Huynh-Feldt correction when epsilon exceeded 0.75 (Girden, 1992). For all statistical analyses, the alpha level (*p*) for determining statistical significance was set at 0.05. Effect sizes were measured using partial eta squared ($\eta_{p}^{2}$) for all ANOVA analyses, and pairwise comparisons were assessed using Cohen's *d* as the effect size metric (Cohen, 1988).

## Results

### Drowning Detection Performance

#### Main Effects

**Figure 2:**
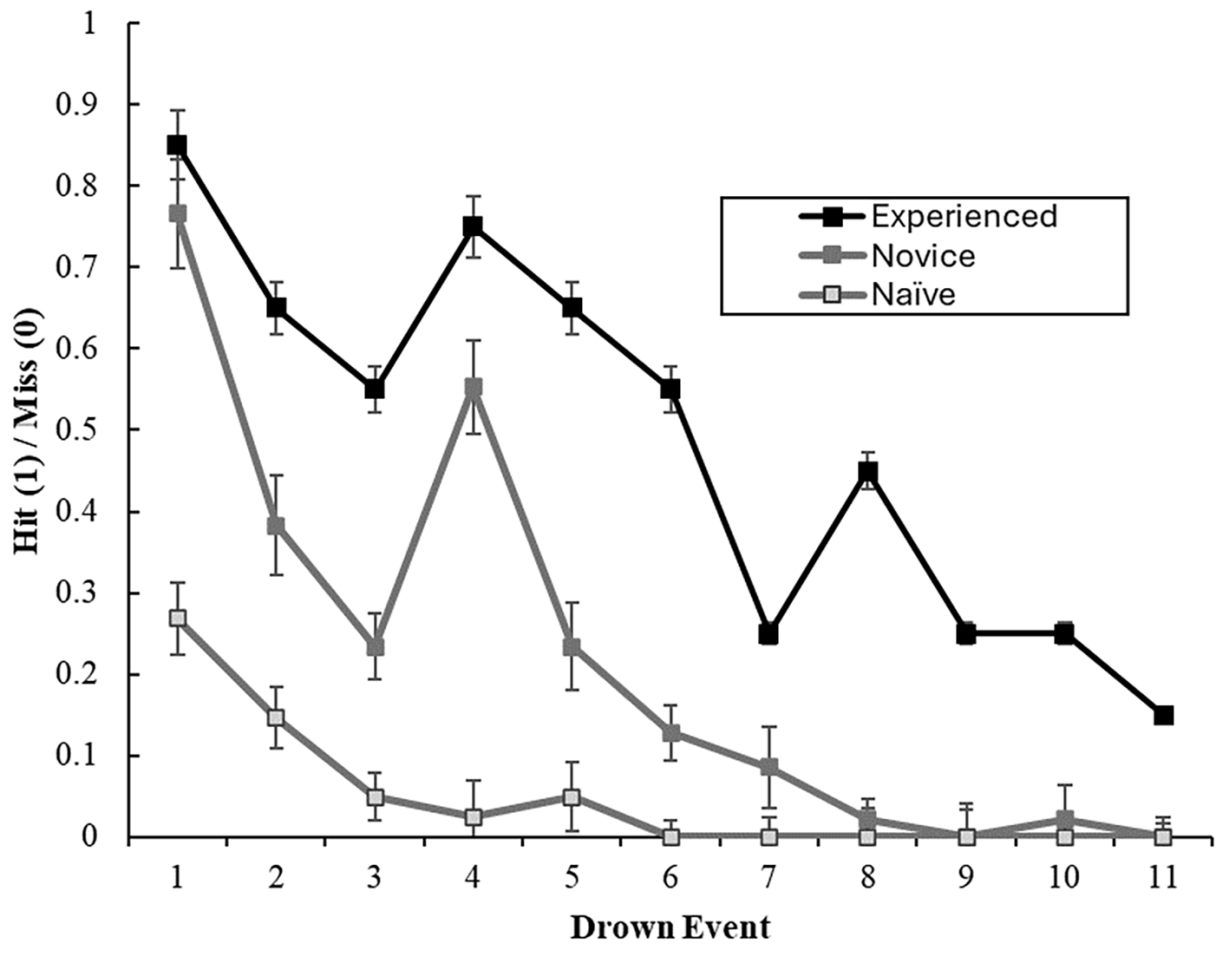
The Influence of Experience and Time on Drowning Detection Performance (With *SE* Bars) *Note.* Drown events occurred every five minutes (e.g., 1 = 5 minutes, 2 = 10 minutes…).

There was a significant main effect of lifeguard experience on total drowning detection performance, *F*(2, 105) = 222.078, *p* < .001, $\eta_{p}^{2}$ = .809. The experienced group performed 26.6%, 95% CI [21.6%, 31.6%] greater than intermediate, *t*(66) = 13.028, *p* < .001, *d* = 1.254, and 43.8%, 95% CI [38.7%, 48.8%] greater than the novice group, *t*(60) = 20.989, *p* < .001, *d* = 2.020. Further, intermediate lifeguards also demonstrated an overall 17%, 95% CI [13.2%, 21.1%] advantage over the novice group, *t*(87) = 10.513, *p* < .001, *d* = 1.012. There was a significant main effect of performance across time points, *F*(6.817, 715.753) = 28.266, *p* < .001, $\eta_{p}^{2}$ = .212. On average drowning detection performance began to deteriorate as time progressed (see Figure 2).

#### Interaction Effects

Time had a 2-way significant interaction effect with lifeguard experience, *F*(13.633, 715.753) = 4.635, *p* < .001, $\eta_{p}^{2}$ = .081. Three separate post hoc one-way ANOVAs demonstrated significant differences in drowning detection performance across time points for experienced, *F*(10, 190) = 4.957, *p* < .001, $\eta_{p}^{2}$ = .207, intermediate, *F*(10, 460) = 24.982, *p* < .001, $\eta_{p}^{2}$ = .352, and novice groups, *F*(10, 400) = 6.991, *p* < .001, $\eta_{p}^{2}$ = .149. From Time Point 1, experienced lifeguards demonstrated a significant decline in performance at Time Point 7, *t*(19) = 5.756, *p* < .001, whilst the intermediate group demonstrated an immediate significant decline in performance at Time Point 2, *t*(46) = 5.632, *p* < .001. Despite the significant interaction for novices, follow up *t*-tests showed no significant differences in drowning detection performance when comparing the timepoints in order of time. Novices reached a floor effect at Time Point 6, where no drowning scenarios were detected, which extended for the remainder of the task. At Time Point 1, novices significantly underperformed compared to the experienced, *t*(60) = 6.593, *p* < .001, and intermediate groups, *t*(87) = 7.199, *p* < .001.

### Number of Fixations

#### Main Effects

**Figure 3:**
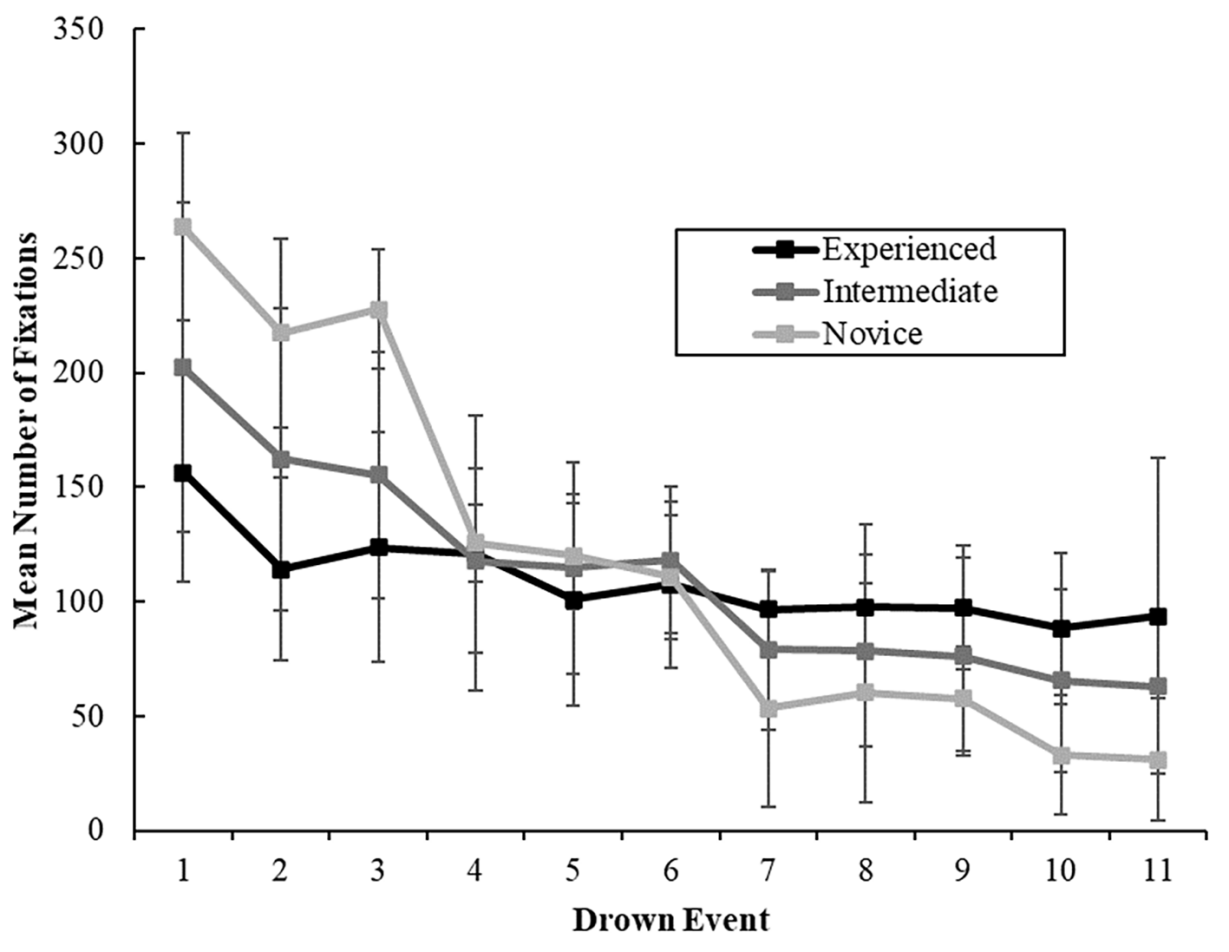
The Influence of Experience and Time on Number of Fixations (With *SE Bars*) *Note.* Drown events occurred every five minutes (e.g., 1 = 5 minutes, 2 = 10 minutes…).

There was a significant main effect of lifeguard experience on total number of fixations, *F*(2, 105) = 7863.457, *p* < .001, $\eta_{p}^{2}$ = .124. The experienced group had fewer fixations, *M* = 1196.85, *SD* = 94.28, than the novice group, *M* = 1300.07, *SD* = 99.98, *t*(60) = -3.506, *p* < .001, *d* = 0.337. Likewise, the intermediate group, *M* = 1232.75, *SD* = 119.33, demonstrated significantly fewer fixations compared to the novice group, *t*(87) = -2.918, *p* < .05, *d* = 0.281. The experienced group did not demonstrate a significant difference compared to the intermediate group, *t*(66) = -1.245, *p* > .05. There was a significant main effect of number of fixations across time points, *F*(5.920, 621.623) = 119.725, *p* < .001, $\eta_{p}^{2}$ = .533. On average the total number of fixations between drowning events decreased as time progressed (see Figure 3).

#### Interaction Effects

Time had a 2-way significant interaction effect with group, *F*(11.840, 621.623) = 18.190, *p* < .001, $\eta_{p}^{2}$ = .257. Three separate post hoc one-way ANOVAs demonstrated significant differences in number of fixations across time points for experienced, *F*(10, 190) = 8.199, *p* < .001, $\eta_{p}^{2}$ = .301, intermediate, *F*(10, 460) = 42.045, *p* < .001, $\eta_{p}^{2}$ = .478, and novice groups, *F*(10, 400) = 158.263, *p* < .001, $\eta_{p}^{2}$ = .798. From Time Point 1, experienced lifeguards demonstrated a significant decline in number of fixations at Time Point 5, *t*(19) = 4.066, *p* < .05. For intermediate and novice groups, there was an immediate significant decline in number of fixations at Time Point 2, *t*(46) = 4.507, *p* < .01, and *t*(40) = 4.856, *p* < .001, respectively.

### Fixation Duration

#### Main Effects

**Figure 4:**
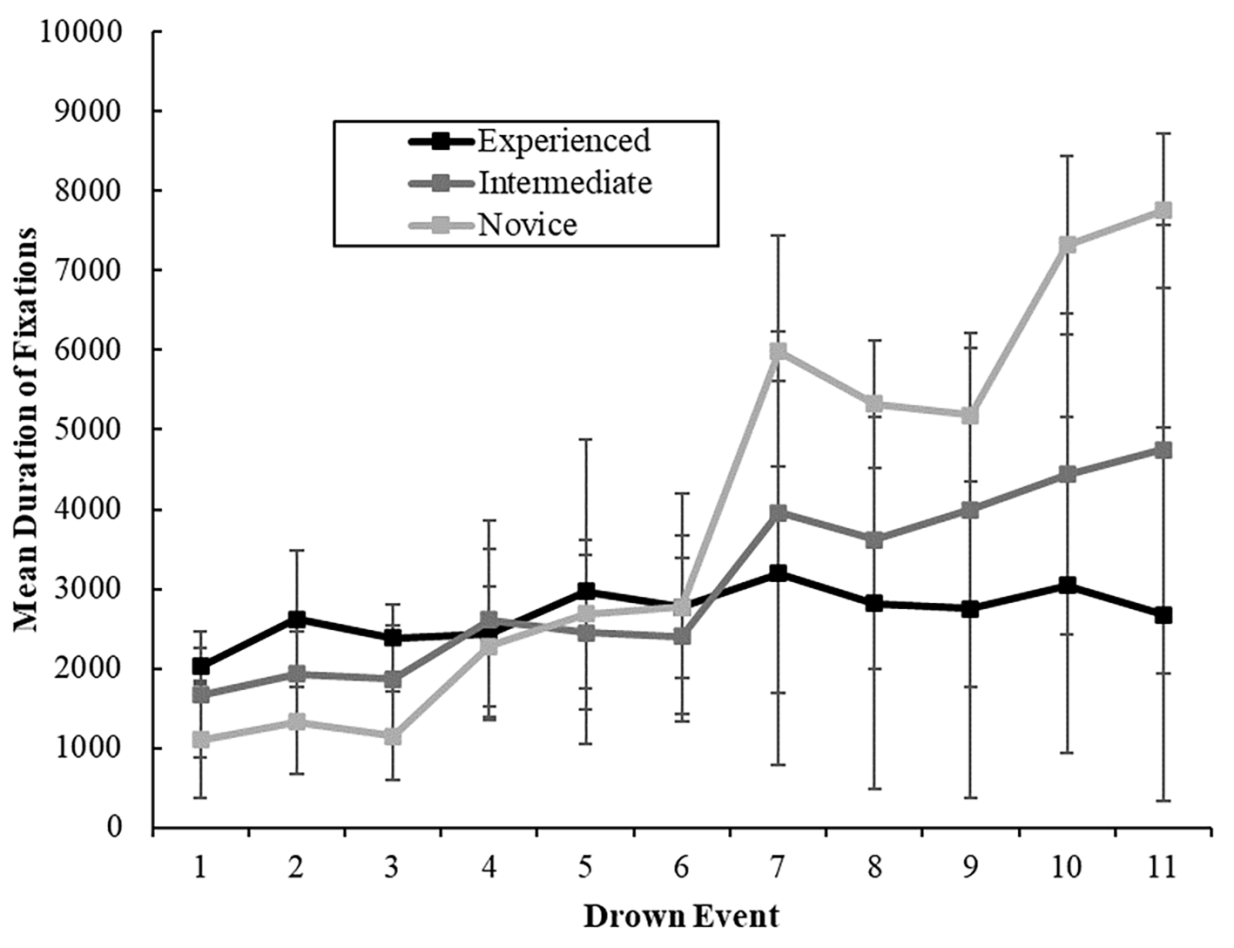
The Influence of Experience and Time on the Average Duration of Fixations (in Milliseconds) (With *SE* Bars) *Note.* Drown events occurred every five minutes (e.g., 1 = 5 minutes, 2 = 10 minutes…).

There was a significant main effect of lifeguard experience on average fixation duration, *F*(2, 105) = 38.305, *p* < .001, $\eta_{p}^{2}$ = .422. The experienced group, *M* ms = 2458.74, *SD* = 530.83, had longer fixation durations on average compared to the novice group, *M* ms = 3243.71, *SD* = 327.35, *t*(60) = 7.775, *p* < .001, *d* = 0.748, but not the intermediate group, *M* ms = 2501.32, *SD* = 692.60, *t*(66) = 2.416, *p* > .05. Further, intermediate lifeguards demonstrated longer average fixation durations compared to the novice group, *t*(87) = 6.905, *p* < .001, *d* = 0.664. There was a significant main effect of average fixation durations across time points, *F*(5.103, 535.793) = 284.152, *p* < .001, $\eta_{p}^{2}$ = .730). On average the mean duration of fixations between drowning events increased as time progressed (see Figure 4).

#### Interaction Effects

Time had a 2-way significant interaction effect with experience, *F*(10, 1050) = 13.381, *p* < .001, $\eta_{p}^{2}$ = .203. Three separate post hoc one-way ANOVAs demonstrated significant differences in fixation duration across time points for experienced, *F*(10, 190) = 95.600, *p* < .001, $\eta_{p}^{2}$ = .834, intermediate, *F*(10, 460) = 111.683, *p* < .001, $\eta_{p}^{2}$ = .708, and novice groups, *F*(10, 400) = 140.457, *p* < .001, $\eta_{p}^{2}$ = .778. For the experienced lifeguards there was no statistically significant differences in the mean duration of fixations at time between Time Point 1 and 11, *t*(19) = 11.898, *p* > .05. From Time Point 1, the intermediate group had a significant increase in the mean duration of fixations at Time Point 7, *t*(46) = 6.332, *p* < .001. Likewise, the novice group saw a significant increase in mean duration of fixations at Time Point 5, *t*(40) = 6.574, *p* < .001.

### Blink Duration

#### Main Effects

There was a significant main effect of lifeguard experience on average blink duration, *F*(2, 105) = 3.875, *p* < .05, $\eta_{p}^{2}$ = .069. The experience group had a shorter average blink duration over the task, *M_ms_* = 33.88, *SD* = 2.28, than the novice group, *M_ms_* = 35.57, *SD* = 2.40, *t*(60) = -2.730, *p* < .05, *d* = 0.022, but not the intermediate group, *M_ms_* = 34.78, *SD* = 2.14, *t*(66) = 1.480, *p* > .05. Likewise, the intermediate group did not demonstrate a significant difference to the novice group, *t*(87) = -1.636, *p* > .05. There was no significant main effect of average blink duration across time points, *F*(10, 1050) = 1.029, *p* > .05.

#### Interaction Effects

No 2-way interaction effects were observed between time and experience group, *F*(20, 1050) = 0.828, *p* > .001.

## Discussion

The study aimed to explore the gaze patterns exhibited by lifeguards with varying levels of experience while performing a drowning detection task specific in duration to their profession. We postulated that performance would decline with time due to factors such as fatigue and reduced vigilance. Our results supported this, as we observed a decrease in detection rates over time for the experienced and intermediate groups. Furthermore, building on earlier studies, we hypothesized that experienced lifeguards would outperform those with less experience, and this was borne out by the data. Specifically, the experienced group detected significantly more drowning events than their intermediate and novice counterparts. We posited that changes in detection over time and group differences would be reflected in the lifeguards' gaze behaviours. Our findings revealed alterations in fixation number for all groups as time elapsed, although fixation duration only varied over time for the intermediate and novice groups. Interestingly, blink duration was shorter for experienced lifeguards than novices, but this did not differ between experienced and intermediate, or intermediate and novice lifeguards, and there was no change in blink duration over time.

In the context of lifeguard vigilance, our study showed that drowning detection rates decreased over time for the experienced and intermediate groups, but not for the novice group. However, the experienced group did not exhibit a significant decline in detection performance until Time Point 7 (35 minutes into the task), whereas the intermediate group showed a decline at Time Point 2 (five minutes into the task). It is possible that the novice group did not experience a decline over time due to their low initial hit rate (0.27 hit rate), which may have limited their opportunity to deteriorate further. These findings suggest that experienced lifeguards possess superior attentional control, enabling them to maintain an attentional advantage for a longer period than their less experienced counterparts. This may be indicative of executive function skills that enable prolonged endogenous control mechanisms, thereby enhancing goal-directed behaviour and reducing vigilance decrement. This may enable experienced lifeguards to resist mind-wandering (Killingsworth & Gilbert, 2010) or ignore irrelevant aspects of the display (Vansteenkiste et al., 2021) for longer periods. The findings have practical implications for lifeguard training and practice, including regular breaks during pool observation and pairing experienced lifeguards with less experienced ones to counter the accelerated decline in performance. Furthermore, such findings suggest that lifeguards would benefit from training their executive function skills to enhance their resilience to fatigue and boredom.

Our study demonstrated that experienced lifeguards had a higher drowning detection rate than intermediate and novice lifeguards over the course of the 60-minute task. This finding is consistent with previous research on lifeguarding (Sharpe et al., 2023) and supports the recommendation to train lifeguards to enhance their hazard detection skills and accelerate their expertise in this area. Our investigation revealed that changes in detection performance over time were associated with changes in gaze behaviour. Notably, experienced lifeguards maintained their number of fixations and mean fixation duration throughout the task until Time Point 5 (25 minutes), suggesting that their attention became less exhaustive after approximately 20–25 minutes. In contrast, intermediate and novice lifeguards showed significant decreases in the number of fixations at Time Point 2, and significant increases in fixation duration at Time Points 7 and 5, respectively. These findings suggest that intermediate and novice lifeguards have a less robust gaze process than experienced lifeguards.

In the context of lifeguard expertise, our research has uncovered noteworthy distinctions among groups in terms of eye movement variables. Specifically, our findings indicate that seasoned lifeguards demonstrate a more sophisticated search pattern compared to novices, characterized by fewer fixations of extended duration. This observation aligns with prior research suggesting that prolonged fixation duration enhances information extraction capabilities (Mann et al., 2007). Given the intricacy of the visual display employed in our study, consisting of 32 items, as opposed to the parameters explored in existing literature, it is plausible that there exists a set size beyond which a serial search pattern may no longer be optimal. This scenario could prompt lifeguards to employ alternative mechanisms, such as more foveal spots (i.e., visual processing utilizing the fovea), gaze anchors (i.e., peripheral vision-based information processing), or visual pivots (i.e., distance-optimized points between relevant cues facilitating saccade initiation; refer to Vater et al., 2020 for detailed discussion). These mechanisms may contribute to longer fixation durations, potentially enhancing lifeguards' hazard perception abilities. Furthermore, our data indicates no discernible differences in the number of fixations or fixation duration between experienced and intermediate lifeguard groups. However, both experienced and intermediate groups exhibited fewer fixations of longer duration than the novice group, suggesting that accumulated experience may influence lifeguards' inclination to concentrate their attention in a specific area, possibly as a foveal spot, anchor, or pivot. Subsequent research is warranted to ascertain the optimal set size at which a more refined search pattern becomes imperative.

In our investigation of blink duration, a notable contrast emerged between experienced and novice lifeguards, with the former displaying shorter blink durations. This observation holds significance, given that blink duration has been previously associated with fatigue (Maffei & Angrilli, 2018; Paprocki & Lenskiy, 2017). The implication is that experienced lifeguards may be less susceptible to fatigue while engaged in the surveillance of a water-related environment. This finding aligns cohesively with our earlier discovery that experienced lifeguards exhibit longer fixation durations than their novice counterparts, a characteristic that may contribute to heightened hazard detection by mitigating the impact of fatigue on attentional processes.

The observed declines in detection performance over time appear to be related to changes in gaze behaviour. Specifically, lifeguards tend to refine their gaze strategy by using fewer fixations of equal duration (in experienced lifeguards) or longer duration (in intermediate and novice lifeguards) as time passes. While such changes may reflect the vigilance decrement, they may also represent a direct strategy employed by lifeguards when engaging in long-duration hazard detection tasks. Notably, detection performance among experienced lifeguards did not decline until Time Point 7 (35 minutes) despite changes in fixation behaviour occurring earlier, at Time Point 4 (20 minutes) for number of fixations and Time Point 5 (25 minutes) for fixation duration. This suggests that gaze behaviour and detection performance are not tightly linked. The implications of these findings suggest that vigilance impacts performance, possibly through direct changes in gaze strategy. To further understand the impact of gaze behaviour on detection performance, future research should employ long-duration tasks (exceeding 35 minutes).

### Implications and Future Directions

Upon closer scrutiny of the data, it becomes apparent that both experienced and intermediate lifeguards may undergo a vigilance decrement, albeit occurring later in the task for more seasoned lifeguards. To our knowledge, this study is the first to illuminate the extent of lifeguard performance decline in tasks extending beyond 35 minutes. This finding holds significant implications, suggesting that lifeguards should incorporate regular breaks or rotate job responsibilities to forestall detection performance from falling below acceptable levels (Helton & Russell, 2015). Such findings may provide tentative support for the recommendations suggested by lifeguard qualifications (i.e., rotating lifeguard positions every 15, 20, or 30 minutes), and justifications for lifeguards not spending more than 30 minutes in one static position. Furthermore, our findings suggest that experience may enable lifeguards to preserve executive function skills, enhancing endogenous control mechanisms and improving goal-directed behavior for detecting hazards over prolonged periods. Given that the turnover rate of lifeguards is typically shorter than in most professions, investigating efficient means to upskill individuals quickly, rather than relying on extended periods of active exposure, could be a valuable avenue for further exploration (see Sharpe et al., 2023 for discussion). Irrespective, lifeguard organisations may wish to invest further resources in exploring the negative outcomes associated with task duration on lifeguard performance, domain specific training (e.g., virtual reality; see Lim et al., 2023), or methods for mediating the such consequence (e.g., detection systems; see Jalalifar et al., 2024).

The present study has demonstrated that the implementation of a more comprehensive gaze strategy resulted in the highest levels of success. However, maintaining such a strategy over time presents a key challenge. To address this challenge, future research may explore the trainability of functions, such as working memory capacity (e.g., Sharpe et al., 2024), to enable novice and intermediate lifeguards to produce exhaustive gaze behaviour, while supporting experienced lifeguards in maintaining their gaze strategy over time. Interestingly, our findings have also revealed differences between groups in terms of gaze strategy. While previous studies in the lifeguard literature have produced mixed results regarding differences between expert and novice lifeguards, the task adopted from Sharpe et al. (2023), which lasted 60 minutes, suggests that differences in search rate may only become apparent when tasks are of longer duration. For instance, in a 45-minute in situ surveillance task, Vansteenkiste et al. (2021) found that fixation duration of experienced lifeguards was longer and more variable than that of novice lifeguards. In contrast, studies using tasks ranging from 29 seconds (Laxton & Crundall, 2018; Laxton, Crundall, et al., 2021; Laxton, Guest, et al., 2021) to 12 minutes (Page et al., 2011) to 20 minutes (Smith et al., 2020) found no differences between expert and novice lifeguards. This suggests that task duration should be taken into consideration when examining gaze behaviour.

### Conclusion

This study aimed to explore variations in gaze behaviour among individuals with different levels of lifeguarding experience during a drowning detection vigilance task. Our preliminary findings suggest that both experienced and intermediate lifeguards exhibit a decline in drowning detection rates as the task progresses. Notably, disparities in gaze behaviour seemed to mirror this performance deterioration, evident in the reduction of fixations across the task for all lifeguards. Individuals with more extensive lifeguarding experience demonstrated a sustained higher average performance and maintained a greater number of fixations for an extended period. Additionally, these experienced lifeguards sustained a consistent fixation duration throughout the entire task, distinguishing them from their less experienced counterparts. These preliminary observations tentatively imply that experienced lifeguards may possess an attentional advantage during vigilance tasks. These findings suggest promising avenues for future research, emphasizing the importance of lifeguard literature in focusing on tasks that accurately simulate the lifeguarding environment, such as vigilance tasks. Further exploration in this direction could enhance our understanding of the factors influencing lifeguard performance and inform training protocols accordingly.

## Supplementary Materials

The Supplementary Materials contain the raw data, analysis code, and additional materials for this study (see Sharpe & Smith, 2024S).



SharpeB. T.
 (2024S). Influence of vigilance performance on lifeguard gaze behaviour
[Research data, code, and materials]. PsychOpen. https://osf.io/9hcg6
10.5964/ejop.12121PMC1163870139678921

## Data Availability

All research data and materials to replicate the findings of this study are publicly available (see Sharpe & Smith, 2024S)
